# An Account of Some Extraordinary Symptoms Occasioned by Worms; with Farther Remarks on Hydrocephalus Internus

**Published:** 1783

**Authors:** 


II. *An account of ſome extraordinary ſymptoms occaſioned by Worms; with farther remarks on Hydrocephalus internus. By Mr.* Edward Wier, *ſurgeon at Halifax in Nova Scotia. Communicated in a letter to Mr.* James Simpſon, *ſurgeon to the Magdalen Hoſpital in London, and by him to Dr.* Simmons.

THE following caſe appears to me to be of a very intereſting nature, as every ſymptom and circumſtance attended it which are commonly obſerved in the hydrocephalus internus. The ſame method of cure was followed which has been found uſeful in that diſeaſe; but the termination of the complaint clearly proved, I think, that the cauſe was miſtaken, though not improperly treated, and that the ſymptoms of worms and thoſe of a dropſy of the brain are ſo nearly alike as to be with the greateſt difficulty diſtinguiſhed; or, perhaps, that hydrocephalus is leſs frequent than is generally ſuppoſed.

The caſe of hydrocephalus I formerly communicated to the public through the channel of the London Medical Journal †, ſeemed to have

† See p. 78.

been as diſtinctly marked, by its ſymptoms, as any diſeaſe could be. In the hiſtory of that caſe it was obſerved, that the patient diſcharged an almoſt incredible number of fine white worms of about the ſize of a cambrick needle, ſhortly after which the ſymptoms began to abate. As worms were never ſuſpected to be the cauſe of the ſymptoms in that caſe, the circumſtance of their being diſcharged under the free uſe of calomel was not laid much ſtreſs on; and had it not been for the very ſingular circumſtances which will be found related in the following caſe, I ſhould perhaps never have been led to ſuppoſe worms capable of producing the ſymptoms of which I ſhall now give an account.

John Stevens, aged two years and two months, had been a remarkable healthy robuſt child till the latter end of October 1782, when he was ſuddenly attacked with vomiting and cried out as if in great pain. His head was violently drawn towards his back, and in a few hours he was obſerved to have a high fever. His mother applied for no medical aſſiſtance till the 4th of November, when I was deſired to ſee him. I found him with his head drawn backwards, and frequently ſcreaming as mentioned before. The pupils of his eyes were much dilated, and the the light seemed to give him great uneasiness. His pulse were 140 in a minute; his breathing was short and quick; his skin hot and dry; and he was observed to be frequently picking his nose, as if it had been much irritated. The vomiting had been almost constant from the first attack of the disease, till about four and twenty hours before I saw him.

This disorder seemed to be so clearly marked as a dropsy of the brain, that I did not hesitate to consider and treat it as such; and the great advantage I had but a few months before derived from a free use of calomel in a similar complaint led me to adopt a similar mode in the present case.

I accordingly ordered three grains of calomel, mixed with sugar, to be administered every four hours; and as the bowels were loaded, I directed a clyster to be immediately thrown up.

The day following (Nov. 5.) he had taken eighteen grains of calomel. The clyster had procured one very fœtid oily stool. The symptoms were nearly as before. I now increased the dose of calomel to four grains every four hours.—The remainder of the case I shall give as written at the time in my diary.

Nov. 6th. Has taken twenty-four grains of calomel ſince yeſterday, and has had three ſlimy fœtid ſtools.—Symptoms not abated.

7th. His fever is rather abated, but his pulſe is ſtill at 120—appears very heavy and dull—the dilatation of the pupils is increaſed, he has a wild ſtaring countenance, and his head is ſtill bent back—has perſevered in the uſe of the calomel, which has no apparent effect on the mouth—has had two ſtools, and paſſed nine very large worms (*lumbricales*).

8th. Countenance, eyes, and head as yeſterday. In the courſe of this day the child has ſpoken ſeveral times, and has ſhewn ſome degree of returning reaſon, but in general when moved hangs his head and limbs as if under a general paralyſis—has taken twenty-four grains of calomel ſince yeſterday and has had two greeniſh coloured ſtools with two large worms—urine ſtill deficient—pulſe 100—the fever regularly returns every night though not with ſo much violence as it did before he took the calomel—ſkin very dry—the patient takes no nouriſhment of any kind—repeat the calomel as before.

9th. Yeſterday afternoon he was ſeveral times in danger of ſuffocation from a ſtoppage in his throat, till at length a worm was diſcharged from from his mouth, and the complaint in his throat ceafed. Has paffed a very reftlefs night—pulfe 90—has had two green ftools, in one of which were nine worms—urine in a very fmall quantity—fever moderate—the head begins to regain its natural pofition—pupils lefs dilated—the patient lies in a heavy fleepy ftate, but when fpoke to opens his eyes, and appears fenfible—repeat the calomel.

10th. He has had three ftools fince yefterday. The firft contained one, the fecond two, and the third nine worms, all alive—the pupils are of a moderate fize, but his countenance appears very wild—pulfe 95—he has a frequent glow of red in one cheek, while the other is pale, and the top of his head very cold—no effect of the calomel is yet to be remarked upon the mouth though he has conftantly fwallowed the quantities mentioned from day to day—he ftill retains his fenfes, though he never attempts to fpeak till firft fpoken to—repeat the calomel as before.

11th. Refted well laft night——this morning pulfe 85——has had two very fœtid flimy ftools, in one of which was a large worm——urine ftill deficient.

12th. Every ſymptom nearly as yeſterday, excepting his having diſcharged more urine than uſual during the laſt twenty-four hours—he has had one ſtool with three worms in it—the vomiting has returned in a conſiderable degree—pulſe 95 and very ſmall—repeat the calomel.

13th. His eyes have a more natural appearance than at any time ſince the attack—the vomiting is abated, but he ſtill takes no nouriſhment—his mouth not in the leaſt affected with the mercury—pulſe 90—I have obſerved for ſeveral days paſt the child very ſuſceptible of cold, which is known by an almoſt conſtant deſire of more covering—he has had two ſtools ſince yeſterday; in the firſt were two, and in the laſt thirteen worms. They were all large and the greater part of them alive.

14th. Has paſſed a reſtleſs night, and has had no ſtools ſince yeſterday—ſymptoms the ſame as yeſterday—repeat the calomel and clyſter.

15th. The patient complains much of cold, and is very weak and languid—takes very little nouriſhment—his eyes, countenance, &c. have quite their natural look.

16th. Had one ſtool yeſterday in the evening, in which were eight worms. I omitted the calomel lomel in the way in which it had been hitherto given, and prefcribed eight grains of calomel and as many of *pulv. jalap.* to be given this morning.

17th. Has had three ftools fince he took the powder; in the firft were ten worms, and in the third one worm—pulfe 95—he is extremely emaciated, and takes but little nourifhment—as he continued to be feemingly in great pain, I adminiftered eight drops of laudanum, which had the defired effect.

19th. Has difcharged one worm without any ftool—the general weaknefs and lofs of appetite feemed now to demand the chief attention, I therefore ordered the bark in the quantity of ten grains every two hours.

20th and 22d. Begins to crave nourifhment—refts well, and appears evidently to be upon the recovery—has had one ftool with a dead worm in it.

23d. Had a ftool this morning with three worms—from this time he daily recovered his appetite, ftrength, and flefh—I continued the ufe of the bark till the end of the month, at which time I difcontinued my vifits as the child was in every refpect in perfect health, and has remained fo ever fince.

That

That this child's diſorder originated from worms, I think, will ſcarcely admit a doubt; and that ſeveral caſes which are recorded and were ſuppoſed to be inſtances of genuine hydrocephalus, and which yielded to the uſe of mercury, might have been nothing more than worm caſes, I think, is extremely probable. Perhaps the many ſimilar caſes which have occurred to very able practitioners, as Dr. Whytt and others, and which proved fatal under the uſe of means which could not have the leaſt tendency to remove worms, were ſuch as would have readily yielded to anthelmintics, at the head of which I have long had reaſon to place calomel. But that we may benefit our patients by the uſe of it, under ſuch circumſtances, very large and frequently repeated doſes appear to be neceſſary. What quantities may be uſed not only with ſafety but advantage, I think, both this and my former caſe tend clearly to evince. Every circumſtance I have related may be depended on, as I paid uncommon attention to the caſe not only with a view to relieve my patient, but at the ſame time, if poſſible, to have it in my power to throw further light on a diſeaſe which has deprived ſo many fond parents of their children. If what I have written ſhould be found to an-

ſwer

ſwer this purpoſe in the ſmalleſt degree, I ſhall ever rejoice in having communicated theſe caſes to the public. I have only to add, that I moſt ſincerely hope that what I have mentioned may be the means of exciting the attention of experienced practitioners to this ſubject, as no one I have ever yet met with appears to have gone further into the idea I have ſuggeſted, than to acknowledge that the ſymptoms of hydrocephalus and thoſe of worms are in many reſpects alike.

The method beſt calculated to throw further light on ſo important a ſubject appears to me to be the following:

Whenever a diſeaſe ſhall occur, which has the ſymptoms which at this day are ſaid to characterize the hydrocephalus internus, let every circumſtance reſpecting the ſymptoms and remedies be faithfully noted down from day to day; let the changes of the former, with the effects of the latter, at the ſame time be remarked. Theſe particulars, with minute accounts of the changes produced in the different ſecretions, the nature of the diſcharge from the different emunctories, with as many particulars of morbid appearances in the bodies of thoſe who die under the diſeaſe, as can any way be obobtained, will in time, perhaps, place the matter beyond a doubt, and enable practitioners with more certainty to diftinguifh the nature of the difeafe, and remove fo formidable a train of complaints.

There is probably too much reafon to fear, that many children have been loft under difeafes which would have yielded to calomel in fufficiently large dofes, merely from proceeding in the too cautious manner moft practitioners do, with this invaluable medicine, particularly in phlegmatic temperaments, as in thefe a confiderable degree of torpor appears to pervade the whole fyftem, while the mucous fecretions are generally very copious, and the folids are for the moft part deficient in degree of irritability. Hence the inteftinal tube is in a manner fo fheathed with mucus, as not readily to be affected with any purge. And that fuch habits are moft predifpofed to generate worms, there can be no doubt, particularly in the infant ftate. ——I have, I think, feen many advantages in fuch habits from the ufe of *myrrh* and *fal mart.* combined, efpecially after calomel has been freely ufed. But in cafes of this fort, unlefs the little patient is put upon a diet of light and eafy digeftion, and every kind of glutinous food is is laid aside, and a considerable degree of exercise recommended, it will, I believe, in general be found to be of but little service to give the most powerful remedies.

